# The association of reactive balance control and spinal curvature under lumbar muscle fatigue

**DOI:** 10.7717/peerj.11969

**Published:** 2021-08-10

**Authors:** Erika Zemková, Alena Cepková, José M. Muyor

**Affiliations:** 1 Department of Biological and Medical Sciences, Faculty of Physical Education and Sport, Comenius University Bratislava, Bratislava, Slovakia; 2 Sports Technology Institute, Faculty of Electrical Engineering and Information Technology, Slovak University of Technology, Bratislava, Slovakia; 3 Centre of Languages and Sports, Faculty of Mechanical Engineering, Slovak University of Technology, Bratislava, Slovakia; 4 Laboratory of Kinesiology, Biomechanics and Ergonomics, Health Research Centre, University of Almería, Almería, Spain

**Keywords:** Compensatory postural responses, Matthiass test, Perturbation-based balance test, Spinal posture, Sørensen fatigue test, Endurance of the hip and back extensor muscles

## Abstract

**Background:**

Although low back fatigue is an important intervening factor for physical functioning among sedentary people, little is known about its possible significance in relation to the spinal posture and compensatory postural responses to unpredictable stimuli. This study investigates the effect of lumbar muscle fatigue on spinal curvature and reactive balance control in response to externally induced perturbations.

**Methods:**

A group of 38 young sedentary individuals underwent a perturbation-based balance test by applying a 2 kg load release. Sagittal spinal curvature and pelvic tilt was measured in both a normal and Matthiass standing posture both with and without a hand-held 2 kg load, and before and after the Sørensen fatigue test.

**Results:**

Both the peak anterior and peak posterior center of pressure (CoP) displacements and the corresponding time to peak anterior and peak posterior CoP displacements significantly increased after the Sørensen fatigue test (all at *p* < 0.001). A lumbar muscle fatigue led to a decrease of the lumbar lordosis in the Matthiass posture while holding a 2 kg load in front of the body when compared to pre-fatigue conditions both without a load (*p* = 0.011, *d* = 0.35) and with a 2 kg load (*p* = 0.000, *d* = 0.51). Also the sacral inclination in the Matthiass posture with a 2 kg additional load significantly decreased under fatigue when compared to all postures in pre-fatigue conditions (*p* = 0.01, *d* = 0.48). Contrary to pre-fatigue conditions, variables of the perturbation-based balance test were closely associated with those of lumbar curvature while standing in the Matthiass posture with a 2 kg additional load after the Sørensen fatigue test (*r* values in range from −0.520 to −0.631, all at *p* < 0.05).

**Conclusion:**

These findings indicate that lumbar muscle fatigue causes changes in the lumbar spinal curvature and this is functionally relevant in explaining the impaired ability to maintain balance after externally induced perturbations. This emphasizes the importance for assessing both spinal posture and reactive balance control under fatigue in order to reveal their interrelations in young sedentary adults and predict any significant deterioration in later years.

## Introduction

In daily life people are repeatedly exposed to a variety of destabilizing perturbations. The center of mass (CoM) during unexpected external perturbations can approach or exceed the limits of stability and fall outside of the base of support. Humans can recover from perturbations with versatile mechanisms using combinations of trunk, upper and lower limb movements (Tokur, Grimmer & Seyfarth, 2020). These recovery movements depend on the perturbation intensity, direction and timing (Tokur, Grimmer & Seyfarth, 2020). Postural and trunk responses to disturbances induced by platform translation differ significantly, such that there is a greater increase of center of pressure (CoP) than CoM displacement (Zemková et al., 2016b). The ability to control rapid balance reactions can be impaired in situations when greater demands are placed on maintenance of postural stability. A systematic review of the evidence by Papa, Garg & Dibble (2015) revealed that muscle fatigue induces clear deteriorations in reactive postural control.

Fatigue is defined as a symptom in which physical and cognitive function is limited by interactions between performance and perceived fatigability (Enoka & Duchateau, 2016). Performance fatigability depends on the contractile capabilities of the muscles involved and the capacity of the nervous system to provide an adequate activation signal for the prescribed task (Enoka & Duchateau, 2016). Perceived fatigability is derived from the sensations that regulate the integrity of the performer based on the maintenance of homeostasis and the psychological state of the individual (Enoka & Duchateau, 2016). These two measures of fatigability normalize the observed fatigue to the demands associated with specific tasks (Enoka & Duchateau, 2016).

Fatigued healthy subjects exposed to external perturbations have shown longer activation latencies (Wilder et al., 1996; Jubany, Danneels & Angulo-Barroso, 2017), an increase in electromyographic amplitude (Dupeyron et al., 2010), reduced muscle activity (Jubany, Danneels & Angulo-Barroso, 2017), and lower trunk muscle co-contractions (Chow et al., 2004) among other changes. These changes most likely contribute to delayed compensatory postural responses to unpredictable stimuli under back muscle fatigue. In particular, delays in muscle activation could decrease the control of the spine possibly leading to chronic back pain (Gill & Callaghan, 1998; Panjabi, 2006; Hodges et al., 2009). A pattern of response to an unexpected external load in people with chronic low back pain (LBP) is closer to that of the fatigued than the non-fatigued healthy subjects (Jubany, Danneels & Angulo-Barroso, 2017). This pertains greater muscle latencies in the activation of the first burst of particular muscles (Radebold et al., 2000; Hodges, 2001; Mehta et al., 2010; Shenoy, Balachander & Sandhu, 2013). Similar temporal alterations in people with chronic LBP and healthy fatigued subjects could allow us to examine postural responses to external perturbations in healthy populations while reducing the eventual consequences for those with LBP.

People with chronic LBP experience excessive fatigability of the back muscles (Mannion et al., 1997; Latimer et al., 1999; Da Silva et al., 2015). This may be a consequence of their sedentary lifestyle which results in markedly decreased force output and fatigue resistance. Higher fatigue in sedentary than physically active people is related to a longer time spent sitting (Engberg et al., 2017) and this can be a major cause of back pain. Lack of exercise induces joint contracture, a constriction or stiffness of joints (Moriyama, 2017). Physical inactivity also causes changes in muscle fibres, which may contribute to muscle stiffness (Herzog et al., 2015). Fatigue-induced reduction in active muscle stiffness increases antagonistic co-contraction to maintain stability resulting in increased spinal compression with fatigue (Granata, Slota & Wilson, 2004). Fatigue-induced reduction in force-generating capacity limits the feasible set of muscle recruitment patterns, thereby restricting the estimated stability of the spine (Granata, Slota & Wilson, 2004). People with LBP use a more rigid strategy to maintain postural stability in comparison with a multisegmental strategy utilized by healthy individuals (Allum et al., 1998; Morasso & Schieppati, 1999; Brumagne, Cordo & Verschueren, 2004). This strategy may lead to instability when demands on postural control increase (Mok, Brauer & Hodges, 2007; Brumagne et al., 2008).

The question remains as to whether there is an association between fatigue-induced changes in reactive postural control and spinal alignment compensating for back muscle fatigue. Lumbar extensor fatigue affects both postural control and joint kinematics during quiet standing (Madigan, Davidson & Nussbaum, 2006). This affect may be ascribed as an alternation in the sensory, motor, and/or central integration and processing components involved in maintaining balance. Both inadequate central integration of sensory information and impaired neuromuscular functions under fatigue can modify feedforward and feedback control of postural sway. However, when individuals are subjected to unpredictable perturbations, anticipation strategies of postural adjustments cannot be applied and corrective postural reactions are used. Perturbations can alter one or more components of the spinal stabilizing system, consisting of the spinal column, the spinal muscles and the neural control unit (Panjabi, 1992). In the presence of muscle fatigue, the effectiveness of these systems involved in postural and spinal stability can be reduced in individuals with a less efficient muscle control system. Determining the influence of back muscle fatigue on reactive balance control and spinal stability in individuals with a predominantly sedentary lifestyle is therefore of great interest. Also, the direct evidence linking reactive postural responses to external stimuli and spinal curvature under back muscle fatigue is yet to emerge.

To address these issues, we investigated (1) the effect of lumbar muscle fatigue on compensatory postural responses to externally induced perturbations and the sagittal spinal curvature in the Matthiass standing posture with an additional load, and (2) the relationship between variables of these active and passive components of core stability prior to and after the back extension endurance test. We hypothesized that (1) lumbar muscle fatigue induced by repetitive arch-ups would have a detrimental effect on compensatory postural responses to unexpected external perturbations and lumbar spine curvature in young sedentary adults, and that (2) there would be a close relationship between these components of core and spinal stability after the Sørensen fatigue test.

## Materials & Methods

### Participants

A group of 38 healthy men, students at a technical university (age 20.4 ± 1.3 y, height 181.1 ± 5.5 cm, and body mass 79.2 ± 8.5 kg) volunteered to participate in the study. They were predominantly sedentary or performed very little physical activity, undergoing mainly obligatory exercise courses at the university or participating in sporting activities at a recreational level (1–2 times a week). According to Booth & Lees (2006) sedentary or physically inactive subjects are defined as those with less than 30 min of daily physical activity, which is the generally agreed threshold level for health benefits as a result of physical activity. Participants in our study met these criteria. They were eligible if they did not report back pain, particularly in the lumbar region. Those who had previously undergone medically invasive procedures for LBP were excluded. Participants were informed of the main purpose of the study and procedures described. Verbal informed consent was provided. All procedures were in accordance with the 1964 Helsinki Declaration and its later amendments. The ethics committee of the Faculty of Physical Education and Sport of the Comenius University Bratislava approved projects Nos. 4/2017 and 1/2020.

### Experimental design

This is a cross-sectional study designed to assess the effect of fatigue on the ability to maintain postural stability after perturbations induced by a 2 kg load release held in front of the body and spine curvature in the Matthiass posture (Matthiass, 1961) while holding a 2 kg load in front of the body. Pre-fatigue conditions included a measurement of the spine curvature in an upright stance and the Matthiass posture without and with an additional load of 2 kg as well as an assessment of compensatory postural responses to externally induced perturbations. Detailed descriptions of the Sørensen fatigue test (Biering-Sørensen, 1984) that examines the endurance of the hip and back extensor muscles, the perturbation-based balance test and the spinal curvature measurement are included in the File S1.

### Statistical analysis

The hypothesis of normality was analysed *via* the Kolmogorov–Smirnov test. A parametric analysis was performed because the data was normally distributed. To test for fatigue-induced changes in the variables of a perturbation-based balance test, a one-way analysis of variance (ANOVA) was used. A one-way ANOVA with repeated measurements for the factor “posture” was also used to compare the spinal curvatures (thoracic and lumbar), the sacral and trunk inclination in a standing posture, in the Matthiass test with and without an additional load prior to the lumbar fatigue test, and in the Matthiass test with a 2 kg load after the lumbar fatigue test. The Wilk’s lambda, Pillai’s trace, the Hotelling trace and Roy’s tests were used to confirm the significance of the repeated multivariate measurements, which all variables obtained similar results. If the main effect of the ANOVA showed a significant *p* value, *post hoc* tests were performed using Bonferroni correction, adjusting the factor to a value of 0.0125 (0.05/4). Cohen’s *d* was used to evaluate effect size for each comparison. An effect size less than 0.2 was considered small, approximately 0.5 was moderate, and greater than 0.8 was large (Cohen, 1988). The statistical power and effect sizes were calculated with the software program G*power 3.1 for Mac OS X (Faul et al., 2007). *A priori* sample size estimate (Cohen, 1988) for an alpha level 0.05, effect size 0.25, and Power (1-*β* error probability) 0.95 was calculated. A total sample size of 36 participants appeared to be necessary to detect the differences among the angular spinal curvatures, and sacral and trunk inclination obtained from different tests. Statistical data was analyzed with the software program IBM SPSS (*v.* 25) with a level of significance of *p* < 0.05. Associations between the variables of the perturbation-based balance test and those of spinal curvature were assessed using Pearsons product moment correlation coefficient (*r*). Data is presented as mean values ± standard deviations (SD).

## Results

Pre to post fatigue changes in variables of the perturbation-based balance test are shown in Table 1. Both the peak anterior and peak posterior CoP displacements and the corresponding time to peak anterior and to peak posterior CoP displacements significantly increased after the Sørensen fatigue test. Accordingly, lumbar muscle fatigue induced by repetitive arch-ups increased significantly total anterior to posterior CoP displacement and corresponding time from peak anterior to peak posterior CoP displacement.

Angle values of the thoracic and lumbar spine, sacral and trunk inclination prior to and after the Sørensen fatigue test are shown in Table 2. Angle values of lumbar lordosis in the Matthiass posture with a 2 kg load were significantly lower after the Sørensen fatigue test when compared to these values in pre-fatigue conditions both without a load (*p* = 0.011, *d* = 0.35) and while holding a 2 kg load (*p* = 0.000, *d* = 0.51). Similarly, angle values of the sacral inclination in the Matthiass posture with a 2 kg load were significantly lower after the Sørensen fatigue test when compared to these values in all other postures (*p* = 0.01, *d* = 0.48). Furthermore, angle values of thoracic spine were significantly higher in the standing than Matthiass posture, independently of the situation (with or without an additional load; pre- or post-lumbar muscle fatigue) (*p* = 0.000, *d* = 0.23). Also angle values of the sacral inclination were significantly higher in the standing than Matthiass posture, independently of the situation (with or without an additional load; pre- or post-lumbar muscle fatigue) (*p* = 0.000, *d* = 0.60). However, angle values of the trunk inclination were significantly lower in the standing than Matthiass posture, independently of the situation (with or without an additional load; pre- or post-lumbar muscle fatigue) (*p* = 0.000, *d* = 0.71). Pairwise comparisons of angle values of thoracic and lumbar spine, sacral and trunk inclination under different postures prior to and after the Sørensen fatigue test are shown in Table 3.

**Table 1:** Parameters of the perturbation-based balance test prior to and after the Sørensen fatigue test.

Bipedal stance on a force plate with eyes open	Pre-test Mean (SD)	Post-test Mean (SD)	*P*-value	Effect size (Cohen’s *d*)
Time to peak anterior CoP displacement (ms)	184.7 (39.0)	230.4 (29.0)	0.000	1.33
Peak anterior CoP displacement (mm)	2.5 (0.9)	4.9 (2.1)	0.000	1.49
Time to peak posterior CoP displacement (ms)	331.2 (85.2)	522.3 (171.7)	0.000	1.41
Peak posterior CoP displacement (mm)	29.3 (4.3)	39.0 (7.2)	0.000	1.64
Time from peak anterior to peak posterior CoP displacement (ms)	146.5 (102.4)	291.8 (176.6)	0.000	1.01
Peak anterior to peak posterior CoP displacement (mm)	26.8 (4.0)	34.1 (7.4)	0.000	1.23

**Table 2:** Angle values of the thoracic and lumbar spine, sacral and trunk inclination prior to and after the Sørensen fatigue test.

	Thoracic spine Mean (SD)	Lumbar spine Mean (SD)	Sacral inclination Mean (SD)	Trunk inclination Mean (SD)
Standing posture	37.34 (10.02)	−27.47 (7.34)	12.97 (5.65)	−1.66 (2.37)
Matthiass posture: Pre-fatigue without a load	31.89 (10.14)	−27.45 (6.14)	10.32 (5.64)	−6.95 (3.07)
Matthiass posture: Pre-fatigue with a 2 kg load	31.42 (11.57)	−28.53 (6.45)	9.97 (5.48)	−8.21 (3.45)
Matthiass posture: Post-fatigue with a 2 kg load	31.29 (12.00)	−25.32 (5.97)	8.24 (5.98)	−6.66 (5.20)

**Table 3:** Pairwise comparisons of angle values of thoracic and lumbar spine, sacral and trunk inclination under different postures prior to and after the Sørensen fatigue test.

		Matthiass posture Pre-fatigue without a load	Matthiass posture Pre-fatigue with a 2 kg load	Matthiass posture Post-fatigue with a 2 kg load
Thoracic spine	Standing posture	^‡^	^‡^	^‡^
	Matthiass posture: Pre-fatigue without a load	–	NS	NS
	Matthiass posture: Pre-fatigue with a 2 kg load	–	–	NS
Lumbar spine	Standing posture	NS	NS	NS
	Matthiass posture: Pre-fatigue without a load	–	NS	^*^
	Matthiass posture: Pre-fatigue with a 2 kg load	–	–	^‡^
Sacral inclination	Standing posture	^‡^	^‡^	^‡^
	Matthiass posture: Pre-fatigue without a load	–	NS	^†^
	Matthiass posture: Pre-fatigue with a 2 kg load	–	–	^*^
Trunk inclination	Standing posture	^‡^	^‡^	^‡^
	Matthiass posture: Pre-fatigue without a load	–	^†^	NS
	Matthiass posture: Pre-fatigue with a 2 kg load	–	–	NS

**Notes.**

* *p* ≤ 0.05.

† *p* ≤ 0.01.

‡ *p* < 0.001.

NS not significant

Furthermore, there were significant correlations (all *p* < 0.05) between angle values of the lumbar spine in the Matthiass posture with an additional load of 2 kg and peak posterior CoP displacement (r =−0.631), time to peak posterior CoP displacement (r =−0.528), peak anterior to peak posterior CoP displacement (r =−0.520) and time from peak anterior to peak posterior CoP displacement (r =−0.631) after the Sørensen fatigue test but not in pre-fatigue conditions. However, only small to moderate correlations were found between values of the perturbation-based balance test and angle values of the thoracic spine, sacral inclination and trunk inclination in all remaining postures regardless of lumbar muscle fatigue.

## Discussion

Lumbar muscle fatigue impairs compensatory postural responses to perturbations induced by a 2 kg load release. Both the peak anterior and peak posterior CoP displacements and the corresponding time to peak anterior and peak posterior CoP displacements significantly increased after the Sørensen fatigue test. These findings are in agreement with previous studies that have reported impairments of postural control under fatigue, especially when involving lumbar trunk muscles (Davidson, Madigan & Nussbaum, 2004; Madigan, Davidson & Nussbaum, 2006; Vuillerme, Anziani & Rougier, 2007; Lin et al., 2009). When individuals are subjected to unexpected trunk perturbations, greater demand is placed on the neuromuscular system, which contributes to overall balance control and spinal stabilization. Therefore, impairment of neuromuscular functions under back muscle fatigue (Allison & Henry, 2001; Granata, Slota & Wilson, 2004; Boyas & Guével, 2011; Monjo, Terrier & Forestier, 2015) is believed to reduce the capability to compensate for externally induced postural perturbations. Compensatory mechanisms involving feedforward or feedback postural control may be not sufficient to adjust postural stability when subjected to unpredictable perturbations under fatigue. Trunk muscle fatigue alters anticipatory postural adjustments (Allison & Henry, 2002) as well as the ability to recover from perturbations (Davidson et al., 2009). Proactive changes involve a slight anterior lean prior to the perturbation, and reactive changes are consistent with a shift toward more of a hip strategy with lumbar extensor fatigue (Wilson et al., 2006).

Although fatigue induces changes in the neuromuscular control of postural balance, the sensorimotor system remains partially efficient when the low back region is fatigued (Abboud et al., 2016). In particular, passive spinal structures are important in sensorimotor control of the spine (Holm, Indahl & Solomonow, 2002). Sensory feedback from passive viscoelastic structures of the spine provides local muscle tension and lumbar spine stability (Holm, Indahl & Solomonow, 2002). These changes in lumbar-stabilizing mechanisms in the presence of muscle fatigue seem to be caused by modulation of lumbopelvic kinematics (Descarreaux, Lafond & Cantin, 2010).

Activity of abdominal muscles is another factor to consider during responses to unexpected postural perturbations under fatigue. Trunk extension up to exhaustion initiates an increase in the activity of the abdominal muscles and in the intra-abdominal pressure as the lower back muscles become fatigued (Essendrop, Schibye & Hye-Knudsen, 2002). However, when the fatigue task consisting of a modified version of the Sørensen endurance test was applied, reflex activity amplitude in the back muscle increased while abdominal reflex activity decreased (Abboud et al., 2016). This finding indicates that an increase in erector spinae activity is sufficient to provide spinal stability and compensate for the effect of back muscle fatigue (Cholewicki, Simons & Radebold, 2000; Andersen, Essendrop & Schibye, 2004).

To prevent the onset of fatigue, the neuromuscular system adapts to new conditions and induces changes in motor strategies (Fuller, Fung & Côté, 2011). Such an adaptation can be observed in changes in muscle activity recruitment patterns and muscle co-contraction (Allison & Henry, 2001). The increase in co-contraction reflected by simultaneously activated antagonists is accompanied by smaller knee and hip joint excursions, indicating an elevated level of articular stiffness (Ritzmann et al., 2016). These changes may be associated with an exaggerated postural rigidity and could cause the delayed and reduced postural reactions that are reflected in the changes in CoP displacement when compensating for sudden postural perturbation (Ritzmann et al., 2016). Thus, in the presence of muscle fatigue, multisegmental postural control (Goodworth, Mellodge & Peterka, 2014; McCaskey et al., 2018) can be shifted to a more rigid posture. The fatigue induced changes in stiffness of the musculoskeletal system (Granata, Slota & Wilson, 2004) could compromise the ability of sedentary individuals to compensate for sudden perturbations. They may experience stiffness when the timing and coordination of the muscle activation is not optimal. Increased trunk stiffness beyond some optimal level may result in altered patterns for maintaining balance after perturbations. This may explain increased CoP displacements when compensating for unexpected stimuli under lumbar spine fatigue.

Fatigue most likely augmented stiffness in the hip extensor muscles (Descarreaux, Lafond & Cantin, 2010). Hip, pelvic, and leg muscles interact with arm and spinal muscles *via* the thoracolumbar fascia, so their contraction can influence tension of the posterior layer (Vleeming et al., 1995). Champagne, Descarreaux & Lafond (2008) observed that the hip extensor muscles tend to fatigue simultaneously with the paraspinal muscles during the Sørensen test. Therefore, fatigue of gluteal and hamstring muscles might also contribute to an impaired reactive balance control after repetitive arch-ups in physically inactive individuals.

Furthermore, the physiological response to such an exercise has to be taken into account, though to a lesser extent. The restriction of blood flow due to high intramuscular mechanical pressure is one of the most important factors in lower-back muscle fatigue (Yoshitake et al., 2001). The increase in cardiorespiratory parameters (e.g., pulmonary ventilation, oxygen uptake, heart rate, cardiac output) may also be assumed, as it has been shown during upper and lower body resistance exercises (Zemková et al., 2010; Zemková & Hamar, 2014). However, these variables have not been evaluated in the present study. Therefore, the contribution of these factors to post-exercise postural stability have yet to be investigated, particularly in sedentary individuals with a low level of physical fitness.

Sedentary behaviour, typical for office workers (Jans, Proper & Hildebrandt, 2007; Ayanniyi, Ukpai & Adeniyi, 2010; Toomingas et al., 2012; Collins & O’Sullivan, 2015; Hadgraft et al., 2015; Saidj et al., 2015) or academics (Hanna et al., 2019), has been widely spreading to young adults. Among those young adults, university students tend to spend most of their time in sedentary activities at school as well as during leisure time. This behaviour leads to reduced muscle strength. Young healthy adults lose 2% of their muscle mass after 28 days of bed rest (Paddon-Jones et al., 2004). This muscle weakening is associated with many detrimental effects, including alterations in motor control of deep trunk muscles, and consequently also changes in postural control strategies (Claeys et al., 2015). This is a plausible contributing factor to LBP (Henry et al., 2006). Subjects with LBP have altered automatic postural coordination, both in terms of magnitude and timing of responses, indicating alterations in neuromuscular control (Henry et al., 2006). It is therefore likely that young sedentary adults are at risk for developing future musculoskeletal complaints. Back muscle fatigue in young healthy subjects impairs the ability to adapt their postural control strategy to the prevailing conditions and may result in a similar postural strategy to that observed in patients with recurrent LBP when postural demands increase (Johanson et al., 2011).

Since a sedentary lifestyle emerges as a potential risk factor for back pain, this highlights the importance of identifying postural deviations from a neutral spine. Normal posture is defined as the line of gravity passing through the external auditory meatus, the bodies of the cervical spine, and the acromion and anterior to the thoracic spine (Kendall et al., 2005). In the present study, the measurement of spinal curvature using the Spinal Mouse® during a common clinical test (Matthiass test) was applied to reveal postural adjustments after lumbar muscle fatigue. We have identified a significant decrease in thoracic kyphosis during the Matthiass test in comparison with a standing posture. When the arm is elevated (in a scapulo-humeral flexion), irrespective of the plane of motion, it affects the shoulder girdle and the thoracic spine (Crosbie et al., 2008). This may be due to the elevation of the upper limbs (with a 90° flexion of the shoulders) and a scapular retraction, which could rectify the thoracic spine (Singla & Veqar, 2017). However, there were no significant changes in angular values of the thoracic spine in both pre-fatigue and post-fatigue conditions. This may be ascribed to a restricted range of motion of the thoracic spine by the rib cage as compared to greater mobility of the lumbar spine (Morita et al., 2014). Moreover, fatigue causes a redistribution of active muscles and a reorganization of multi-joint coordination to stabilize equilibrium (Ritzmann et al., 2016). The postural fatigue appears in the distal prior to the proximal musculature (Ritzmann et al., 2016). Thus, the neuromuscular system takes advantage of a shift from the distal to proximal segment for controlling posture and fall avoidance (Ritzmann et al., 2016). This fact could help us to identify a joint stiffness of the thoracic vertebrae and avoid significant changes in pre- *versus* post-fatigue angular values.

Harmony between the lumbar spine and pelvis is critical for adequate spinal sagittal alignment. When imbalances occur, the sagittal vertical axis moves forward. Compensatory mechanisms, including a decrease in thoracic kyphosis, posterior tilting or rotation of the pelvis, hip extension, and even knee flexion, provide optimal alignment of the spine in order to preserve the appropriate position of the gravity line and horizontal gaze (Fechtenbaum et al., 2016). In the present study, the lumbar curvature showed an increase in lordosis during the Matthiass test with a 2 kg load in pre-fatigue conditions. This is because the subject compensates for the anterior displacement of the center of gravity while holding the load (Betsch et al., 2010; Albertsen et al., 2018a; Albertsen et al., 2018b). The lumbar and hip extensor muscles are fatigued significantly during the Sørensen test, which indicates load sharing between back and hip extensor muscles during this test (Kankaanpää et al., 1998). We have found that the fatigue of these muscles significantly decreased the lumbar lordosis and sacral inclination in the Matthiass test while holding a 2 kg load in front of the body when compared to pre-fatigue conditions with and without an additional load. Since the lumbar extensor muscles were limited (due to fatigue), the pelvis was stabilized by an increased gluteal and hamstring activation. In this sense, the gluteals and hamstrings are retroverters of the pelvis, and this posture is associated with less lumbar lordosis (as has been found in the present study).

The only compensatory mechanism in the pelvis area is pelvis back tilt (also called pelvis retroversion), defined by increases in the pelvis tilt corresponding to the posterior rotation of the pelvis around the femoral heads, similar to that during hip extension. This motion is consecutive to contraction of the hip extensor muscles and results in posterior positioning of the sacrum related to the coxo-femoral heads (Barrey et al., 2013). Specifically with regard to the sacrum inclination, there was a significant decrease of its angular values during the Matthiass test in comparison with the standing posture. This posterior tilt could decrease the lumbar lordosis (Levine & Whittle, 1996), as has been observed in the present study.

Regarding the inclination of the trunk, there was a significant increase in the posterior tilt, which was higher while holding the load in order to compensate for the preceding anterior displacement of the center of gravity when elevating the upper limbs (by flexing the shoulders to 90°) (Singla & Veqar, 2017).

These findings indicate that fatigue of the hip and back extensor muscles induced by the dynamic version of the Sørensen test affects both the reactive balance control and lumbar stability. These findings are in agreement with those by Ghamkhar & Kahlaee (2019) who reported that postural control is altered in asymptomatic individuals following trunk muscle fatigue. Lumbar fatigue impairs the ability to sense a change in lumbar position in patients with LBP as well as healthy control subjects (Taimela, Kankaanpää & Luoto, 1999). The reductions in maximum force and rate of force rises that develop with muscle fatigue can also be assumed to impair core stability, which may be aggravated by the adverse effects of fatigue on proprioception of the spinal posture (Taimela, Kankaanpää & Luoto, 1999). Thus, the fatigue of back extensors has a negative effect on the trunk dynamic stability (Granata & Gottipati, 2008). It may affect both neuromuscular recruitment and spinal control. Such a fatigue induced spinal instability may contribute to lower back disorders (Granata & Gottipati, 2008). In particular, reduced co-contraction resulting from a reduction in abdominal activation under fatigue may compromise spinal stability (Gregory et al., 2008). Reduced co-contraction combined with the increased spinal flexion may increase the risk of sustaining an injury to the lower back (Gregory et al., 2008). Apart from lifting and carrying heavy objects or performing repetitive trunk movements, prolonged sitting (Kallings et al., 2021) and standing (Andersen, Haahr & Frost, 2007) can tire or strain the muscles in the lower back, neck and legs, which can lead to aches and pains. Besides exercises that stretch tight muscles, trunk muscle endurance training is crucial to address postural impairment in chronic spinal musculoskeletal conditions (Ghamkhar & Kahlaee, 2019).

Accordingly, spinal stability may be compromised by insufficient muscle force and inappropriate neuromuscular activation (Descarreaux, Lafond & Cantin, 2010). However, the limitation of the study is that the strength of back muscles was not measured. Adding tests of core muscle strength and power (Zemková et al., 2016a; Zemková & Jeleň, 2019; Zemková, Poór & Pecho, 2019) could provide more information about their role in postural readjustments after externally induced perturbations under fatigue. Furthermore, the issue of an additional load should be addressed in subsequent studies. A ∼2 kg weight was used for the perturbation-related balance tests in previous (Shiratori & Aruin, 2007; Jørgensen et al., 2011; Zemková, Štefániková & Muyor, 2016) and in the present study. Alternatively, a load relative to the subject’s body mass can be used (Betsch et al., 2010; Albertsen et al., 2018a). Nonetheless, we were able to observe pre-post exercise changes in reactive balance control when a load representing ∼2.5% of body mass was used in sedentary individuals. A further limitation is that the level of perceived exertion was not estimated. Nevertheless, a relationship between fatigability and back muscle strength was reported using the Biering-Sørensen test (Adams, Mannion & Dolan, 1999). The Sørensen test, performed until failure in a young healthy population, results in a reduced ability of the trunk extensor muscles to generate maximal force, which indicates that this test is valid for the assessment of fatigue in trunk extensor muscles (Demoulin et al., 2016). Our findings can be applied to sedentary individuals, however additional research is needed to investigate their utilization in physically active subjects for whom higher muscle strength may be assumed and/or people with chronic back pain whose problems are long lasting in comparison with the acute fatigue conditions instituted in our study.

## Conclusions

Lumbar muscle fatigue induced by repetitive arch-ups affects postural responses to external perturbations and decreases both lumbar lordosis and sacral inclination. Close relationship between compensatory postural responses to unpredictable stimuli and angle values of the lumbar spine under lumbar muscle fatigue indicates that they are dependent on each other when greater demands on core and spinal stability are required. In other words, lumbar muscle fatigue causes changes in the lumbar spinal curvature, and this would most likely explain adaptive changes that may impair postural reactive control following external and internal perturbations. The impact of fatigue in the lumbar region on the association of reactive balance control with lumbar lordosis has to be taken into account in sedentary people in whom relatively low but persistent fatigue induced by prolonged sitting may affect back health.

##  Supplemental Information

10.7717/peerj.11969/supp-1 Supplemental Information 1 Supplementary material

10.7717/peerj.11969/supp-2 Supplemental Information 2 Data of variables measured during the perturbation-based balance test

10.7717/peerj.11969/supp-3 Supplemental Information 3 Anthropometric parameters and spinal values of the thoracic and lumbar curvatures, sacral tilt and trunk inclination in degrees (°)

## Additional Information and Declarations

### Data Availability

The following information was supplied regarding data availability: The raw measurements are available in the Supplemental Files.
