# Supplementary material for: The association of reactive balance control and spinal curvature under lumbar muscle fatigue
Source: PeerJ. 2021 Aug 10;9:e11969. doi: 10.7717/peerj.11969 (PMC8362667; doi:10.7717/peerj.11969)
Supplement: Supplemental Information 1 [file peerj-09-11969-s001.docx]

**Supplementary material**

***Dynamic Sørensen fatigue test***

Participants underwent a perturbation-based balance test and measurement of spinal curvature prior to and immediately after the dynamic version of the Sørensen fatigue test (*Biering-Sørensen, 1984; Latimer et al., 1999*), called the repetitive arch-up test (*Demoulin et al., 2006*). Participants laid on the Roman chair in the prone position with the upper edge of the iliac crests aligned with the edge of the chair. Arms were folded across the chest, and the ankles and thighs were fixed to the device. The hips remained fully extended throughout the test. The test started with the upper body sloping downward toward the floor so a concentric contraction of the trunk extensor muscles was needed initially to reach the horizontal position. Participants were asked to raise the upper body upwards to a horizontal position and back down to a 45° angle. They performed each repetition in a cadence of 2-3 seconds. The horizontal position of the upper body was visually monitored by the examiner. Hyperextension was not permitted. Participants were given verbal encouragement during arch-ups. The movement was repeated as many times as possible. Once the movement becomes nonsynchronous or jerky, or did not reach the horizontal level, participants were encouraged once to correct the motion. The test was terminated once they were unable to continue with the tempo of the motion or reported exhaustion. The number of repetitions was recorded. Participants performed 22.7 ± 6.3 repetitions.

***A perturbation-based balance test***

Participants performed a perturbation-based balance test while standing on a force plate with eyes focused on a spot on the wall in front of them at eye level. They were instructed to stand in an upright position with their feet abducted 10° and their heels separated mediolaterally at a distance of 6 cm. They stood barefoot on a force plate with their arms held horizontally forward, a shoulder width apart. They were required to hold a bar in their hands with a 2 kg load fixed to the bar. A signal from the computer triggered a random release of the load over a 5 second period following the initiation of the test, thus the subject received no cues as to when the perturbation would occur. The release of the load produced a sudden change in the external forces acting on the subject, leading to a small anterior and then a larger posterior displacement of the subject's center of pressure (CoP). The perturbation after the load fell caused only a postural sway response, i.e. the subject did not need to take a step to maintain balance. The perturbation was quantified by the maximal anterior and posterior displacement, within one second after the load dropped. The recording started about 5 seconds before a load release and ended 2-3 seconds after the load-drop. Participants held arms horizontally forward with a 2 kg load fixed to the bar in their hands during the test duration.

A series of three trials were conducted in random order. Peak anterior CoP displacement, the time to peak anterior CoP displacement, peak posterior CoP displacement, the time to peak posterior CoP displacement, peak anterior to peak posterior CoP displacement, and the time from peak anterior to peak posterior CoP displacement were registered by using the FiTRO Sway Check system, completed with a special program for a Load Release Balance Test (FiTRONiC, SVK). The system measures the actual force in corners of the force plate and calculates instant position of the CoP (sampling rate: 100 Hz, 12 bit AD signal conversion, resolution of the CoP position: less than 0.1 mm, measuring range: 0-1000 N/s, non-linearity: +/- 0.02% FS, combined error: 0.03%, sensitivity: 2mV/V +/- 0.25%, overload capacity: 150% / sensor). The test-retest reliability of the parameters of the load release balance test is good to excellent, with high values of ICC (0.78–0.92) and low SEM (7.1%–10.7%) (*Zemková, Štefániková & Muyor, 2016*). The test is also sensitive enough to discriminate between physically active and sedentary young, early and late middle-aged adults. The area under the ROC curve >0.80 for these variables indicates good discriminatory accuracy (*Zemková, Štefániková & Muyor, 2016*).

***Spinal curvature measurement***

The sagittal spinal curvature and pelvic tilt of each participant were measured in a normal upright stance, the Matthiass standing posture without an additional load, the Matthiass standing posture with a 2 kg load held in the hands in front of the body prior to and after the Sørensen fatigue test. The measurements in the standing posture were performed with the participants barefoot and wearing underwear, and assuming a straight position standing on the floor with the eyes and ears in line with the horizontal axis, arms relaxed at the side of the body, knees close to the individual full extension, and feet shoulder-width apart. The standing posture evaluated during the Matthiass test was the same but with participant's arms at 90º shoulder flexion. The Matthiass test is a common clinical test used to identify postural alterations and has proved as a valid tool in posture assessment (*Matthiass, 1961;* [*Betsch*](https://pubmed.ncbi.nlm.nih.gov/?term=Betsch+M&cauthor_id=20490872) *et al., 2010; Feng et al., 2017; Feng et al., 2018; Kapitán et al., 2019*). It evaluates the dynamic postural performance in the sagittal plane of upright standing individuals while lifting arms forward to 90º shoulder flexion for 30 seconds (*Albertsen et al., 2018*). A forward hip movement accompanied with lumbar extension is a relevant indicator of impaired posture in this test *(Klee, 1995).* There was a close correlation between increased variables in the Matthiass arm-raising test and higher prevalence of pain in the cervical spine, which indicates that neuromuscular stabilization of the thoracic and lumbar spine is related to the function of the cervical spine *(Kapitán et al., 2019).* The measurements of the thoracic and lumbar curvatures, and sacral and trunk inclination was performed with lifted arms for 30 seconds (the duration of this test) in both situations with and without an additional load. In order to standardize the position of the arms, the examiner verbally corrected deviations from the horizonal plane using an inclinometer (ISOMED, Inc., Portland, OR) located in the back of the hand.

Sagittal spinal curvature and pelvic tilt were measured using a Spinal Mouse (Idiag, Fehraltdorf, Switzerland). The Spinal Mouse is an electronic computer-aided measuring device which measures sagittal spinal range of motion and intersegmental angles in a non-invasive way, a so-called surface-based technique. The device is connected by Bluetooth technology to a standard PC. The Spinal Mouse is guided along the midline of the spine. Two rolling wheels follow the contour of the spine, and distance and angle measures are communicated from the device to a base station positioned 1–2 m away and interfaced to a personal computer. Data is sampled every 1.3 mm as the mouse is rolled along the spine, giving a sampling frequency of approximately 150 Hz. The average total length of the spine is 550 mm and the time required to measure the whole length is 2–4 seconds; thus approximately 423 measurements are made over 3 seconds. This information is then used to calculate the relative positions of the sacrum and vertebral bodies of the underlying bony spinal column using an intelligent, recursive algorithm (*Mannion et al., 2004*). The Spinal Mouse is a valid and reliable device for global spinal angles (*Mannion et al., 2004; Post & Leferink, 2004; Guermazi et al., 2006*) that was documented by the ICC as greater than 0.8 for all spinal parameters evaluated (*Mannion et al., 2004*).

Each subject was tested by the same examiner in a single session. Prior to taking measurements, the main researcher determined the spinous process of C7 (the starting point) and the top of the anal crease (the end point) by palpation and marked the skin surface with a pencil. The Spinal Mouse was guided along the midline of the spine (or slightly paravertebrally, particularly in thin individuals with prominent spinous process) starting at the spinous process of C7 and finishing at the top of the anal crease (approximately S3). For each testing position, the position of the thoracic (T1-2 to T11-12) and lumbar (T12-L1 to the sacrum) spine, the position of the sacrum and the hips (the difference between the sacral angle and the vertical plane), and trunk angle of inclination (the angle subtended between the vertical and a line joining C7 to the sacrum) were recorded. The lumbar curve negative values corresponded to lumbar lordosis (posterior concavity). With respect to the sacral position, a value of 0º represented the vertical position. Thus, a greater angle reflected an anterior sacral inclination and a lower angle (negative values) reflected a posterior sacral inclination. In this line (the trunk angle of inclination), positive values show an anterior trunk flexion, and negative values show a trunk in extension.

**References**

Albertsen IM, Dettmann K, Babin K, Stücker R, Schröder J, Zech A, Hollander K. 2018. Spinal postural changes during the modified Matthiass test in healthy children: interday and interrater reliability of dynamic rasterstereographic measurements. *Der Orthopäde* 47(7):567-573 DOI 10.1007/s00132-018-3558-z.

[Betsch](https://pubmed.ncbi.nlm.nih.gov/?term=Betsch+M&cauthor_id=20490872) M, [Wild](https://pubmed.ncbi.nlm.nih.gov/?term=Wild+M&cauthor_id=20490872) M, [Jungbluth](https://pubmed.ncbi.nlm.nih.gov/?term=Jungbluth+P&cauthor_id=20490872) P, [Thelen](https://pubmed.ncbi.nlm.nih.gov/?term=Thelen+S&cauthor_id=20490872) S, [Hakimi](https://pubmed.ncbi.nlm.nih.gov/?term=Hakimi+M&cauthor_id=20490872) M, [Windolf](https://pubmed.ncbi.nlm.nih.gov/?term=Windolf+J&cauthor_id=20490872) J, [Horstmann](https://pubmed.ncbi.nlm.nih.gov/?term=Horstmann+T&cauthor_id=20490872) T, [Rapp](https://pubmed.ncbi.nlm.nih.gov/?term=Rapp+W&cauthor_id=20490872) W. 2010. The rasterstereographic-dynamic analysis of posture in adolescents using a modified Matthiass test. *European Spine Journal* 19(10):1735-9 DOI 10.1007/s00586-010-1450-6.

Biering-Sørensen F. 1984. Physical measurements as risk indicators for low-back trouble over a one-year period. *Spine (Phila Pa 1976)* 9(2):106-19 DOI [10.1097/00007632-198403000-00002](https://doi.org/10.1097/00007632-198403000-00002).

Demoulin C, Vanderthommen M, Duysens C, Crielaard JM. 2006. Spinal muscle evaluation using the Sorensen test: a critical appraisal of the literature. *Joint Bone Spine* 73(1):43-50 DOI 10.1016/j.jbspin.2004.08.002.

Feng Q, Jiang C, Zhou Y, Huang Y, Zhang M. 2017. Relationship between spinal morphology and function and adolescent non-specific back pain: a cross-sectional study. *Journal of Back and Musculoskeletal Rehabilitation* 30(3):625-33 DOI 10.3233/BMR-160544.

Feng Q, Wang M, Zhang Y, Zhou Y. 2018. The effect of a corrective functional exercise program on postural thoracic kyphosis in teenagers: a randomized controlled trial. *Clinical rehabilitation* 32(1):48-56 DOI 10.1177/0269215517714591.

Guermazi M, Ghroubi S, Kassis M, Jaziri O, Keskes H, Kessomtini W, [Hammouda](https://pubmed.ncbi.nlm.nih.gov/?sort=date&term=Ben+Hammouda+I&cauthor_id=16630669) IB, [Elleuch](https://pubmed.ncbi.nlm.nih.gov/?sort=date&term=Elleuch+MH&cauthor_id=16630669) M-H. 2006. Validity and reliability of Spinal Mouse® to assess lumbar flexion. *Annales de Réadaptation et de Médecine Physique* 49(4):172-7 DOI 10.1016/j.annrmp.2006.03.001.

Kapitán M, Pilbauerová N, Vavřičková L, Šustová Z, Machač S. 2019. Prevalence of musculoskeletal disorders symptoms among Czech dental students. Part 2: The predictive value of digital assessment. *Acta Medica (Hradec Králové)* 62(1):6-11 DOI 10.14712/18059694.2019.39.

Klee A. 1995. Predictive value of Matthiass' arm-raising test. *Zeitschrift für Orthopädie und ihre Grenzgebiete* 133(3):207-213 DOI 10.1055/s-2008-1039439.

Latimer J, Maher CG, Refshauge K, Colaco I. 1999. The reliability and validity of the Biering-Sorensen test in asymptomatic subjects and subjects reporting current or previous nonspecific low back pain. *Spine (Phila Pa 1976)* 24(20):2085-9; discussion 2090 DOI 10.1097/00007632-199910150-00004.

Mannion AF, Knecht K, Balaban G, Dvorak J, Grob D. 2004. A new skin-surface device for measuring the curvature and global and segmental ranges of motion of the spine: reliability of measurements and comparison with data reviewed from the literature. *European Spine Journal* 13(2):122-36 DOI 10.1007/s00586-003-0618-8.

Matthiass H. 1961. Measuring methods of the spine in the diagnostic of spine diseases. Stuttgart, Jena, New York: Gustav Fischer.

Post RB, Leferink VJ. 2004. Spinal mobility: sagittal range of motion measured with the SpinalMouse, a new non-invasive device. *Archives of Orthopaedic and Trauma Surgery* 124(3):187-92 DOI 10.1007/s00402-004-0641-1.

Zemková E, Štefániková G, Muyor JM. 2016. Load release balance test under unstable conditions effectively discriminates between physically active and sedentary young adults. *Human Movement Science* 48:142-52 DOI 10.1016/j.humov.2016.05.002.
