# Supplementary material for: The association of reactive balance control and spinal curvature under lumbar muscle fatigue
Source: PeerJ. 2021 Aug 10;9:e11969. doi: 10.7717/peerj.11969 (PMC8362667; doi:10.7717/peerj.11969)
Supplement: Supplemental Information 2 [file peerj-09-11969-s002.docx]

**Table 1.**

Data of variables measured during the perturbation-based balance test.

| **Pre lumbar fatigue** | | | | | | | |  | **Post lumbar fatigue** | | | | | | | |
| --- | --- | --- | --- | --- | --- | --- | --- | --- | --- | --- | --- | --- | --- | --- | --- | --- |
| **n** | **Attempt** | **T1**  **(ms)** | **P1 (mm)** | **T2 (ms)** | **P2 (mm)** | **T1-2 (ms)** | **P1-2 (mm)** |  | | **Attempt** | **T1**  **(ms)** | **P1 (mm)** | **T2 (ms)** | **P2 (mm)** | **T1-2 (ms)** | **P1-2 (mm)** |
| 1 | 1 | 250 | 4.7 | 380 | 28.0 | 630 | 32.7 |  | | 1 | 230 | 6.0 | 600 | 40.8 | 830 | 46.8 |
|  | 2 | 200 | 4.5 | 430 | 30.6 | 630 | 35.1 |  | | 2 | 220 | 4.9 | 590 | 36.2 | 810 | 41.1 |
|  | 3 | 200 | 3.7 | 420 | 27.7 | 620 | 31.4 |  | | 3 | 210 | 3.3 | 540 | 31.1 | 750 | 34.4 |
| 2 | 1 | 200 | 3.0 | 360 | 30.7 | 560 | 33.7 |  | | 1 | 240 | 5.9 | 580 | 39.7 | 820 | 45.6 |
|  | 2 | 220 | 3.1 | 230 | 33.1 | 450 | 36.2 |  | | 2 | 200 | 3.0 | 580 | 35.9 | 780 | 38.9 |
|  | 3 | 180 | 2.3 | 350 | 33.6 | 530 | 35.9 |  | | 3 | 260 | 5.1 | 330 | 34.5 | 590 | 39.6 |
| 3 | 1 | 180 | 4.0 | 320 | 26.7 | 500 | 30.7 |  | | 1 | 260 | 4.2 | 760 | 53.6 | 1020 | 57.8 |
|  | 2 | 190 | 4.0 | 250 | 29.8 | 440 | 33.8 |  | | 2 | 230 | 4.1 | 730 | 52.0 | 960 | 56.1 |
|  | 3 | 170 | 2.7 | 320 | 31.3 | 490 | 34.0 |  | | 3 | 230 | 3.8 | 710 | 39.8 | 940 | 43.6 |
| 4 | 1 | 190 | 1.5 | 270 | 34.2 | 460 | 35.7 |  | | 1 | 170 | 4.6 | 690 | 39.0 | 860 | 43.6 |
|  | 2 | 190 | 1.6 | 350 | 27.2 | 540 | 28.8 |  | | 2 | 170 | 2.7 | 460 | 37.4 | 630 | 40.1 |
|  | 3 | 160 | 0.9 | 260 | 30.0 | 420 | 30.9 |  | | 3 | 230 | 4.5 | 420 | 36.1 | 650 | 40.6 |
| 5 | 1 | 130 | 1.9 | 300 | 36.6 | 430 | 38.5 |  | | 1 | 230 | 4.4 | 440 | 35.3 | 670 | 39.7 |
|  | 2 | 160 | 3.0 | 290 | 38.8 | 450 | 41.8 |  | | 2 | 200 | 5.0 | 400 | 37.2 | 600 | 42.2 |
|  | 3 | 180 | 2.6 | 290 | 35.1 | 470 | 37.7 |  | | 3 | 230 | 4.1 | 420 | 34.0 | 650 | 38.1 |
| 6 | 1 | 170 | 2.5 | 320 | 28.3 | 490 | 30.8 |  | | 1 | 250 | 3.6 | 470 | 47.6 | 720 | 51.2 |
|  | 2 | 190 | 2.2 | 350 | 27.4 | 540 | 29.6 |  | | 2 | 190 | 4.0 | 410 | 45.2 | 600 | 49.2 |
|  | 3 | 200 | 3.4 | 280 | 27.9 | 480 | 31.3 |  | | 3 | 200 | 3.0 | 390 | 44.3 | 590 | 47.3 |
| 7 | 1 | 180 | 2.5 | 650 | 37.8 | 830 | 40.3 |  | | 1 | 210 | 7.8 | 940 | 50.3 | 1150 | 58.1 |
|  | 2 | 200 | 2.7 | 630 | 42.8 | 830 | 45.5 |  | | 2 | 190 | 6.4 | 910 | 49.6 | 1100 | 56.0 |
|  | 3 | 230 | 3.6 | 620 | 43.8 | 850 | 47.4 |  | | 3 | 230 | 5.7 | 870 | 37.7 | 1100 | 43.4 |
| 8 | 1 | 80 | 1.6 | 500 | 26.5 | 580 | 28.1 |  | | 1 | 200 | 1.6 | 490 | 39.0 | 690 | 40.6 |
|  | 2 | 100 | 2.2 | 460 | 25.9 | 560 | 28.1 |  | | 2 | 200 | 9.4 | 360 | 30.5 | 560 | 39.9 |
|  | 3 | 90 | 2.2 | 440 | 21.8 | 530 | 24.0 |  | | 3 | 190 | 1.0 | 310 | 29.6 | 500 | 30.6 |
| 9 | 1 | 200 | 3.1 | 340 | 30.5 | 540 | 33.6 |  | | 1 | 180 | 4.7 | 560 | 33.9 | 740 | 38.6 |
|  | 2 | 190 | 2.9 | 390 | 27.9 | 580 | 30.8 |  | | 2 | 180 | 5.1 | 560 | 31.4 | 740 | 36.5 |
|  | 3 | 190 | 2.2 | 350 | 28.2 | 540 | 30.4 |  | | 3 | 210 | 4.3 | 490 | 27.0 | 700 | 31.3 |
| 10 | 1 | 180 | 4.1 | 300 | 30.9 | 480 | 35.0 |  | | 1 | 250 | 4.2 | 440 | 41.6 | 690 | 45.8 |
|  | 2 | 210 | 3.3 | 230 | 31.1 | 440 | 34.4 |  | | 2 | 250 | 3.9 | 420 | 40.8 | 670 | 44.7 |
|  | 3 | 180 | 3.1 | 250 | 30.0 | 430 | 33.1 |  | | 3 | 200 | 3.3 | 380 | 35.2 | 580 | 38.5 |
| 11 | 1 | 170 | 3.3 | 370 | 25.8 | 540 | 29.1 |  | | 1 | 230 | 6.3 | 940 | 43.2 | 1170 | 49.5 |
|  | 2 | 190 | 2.6 | 300 | 22.6 | 490 | 25.2 |  | | 2 | 210 | 5 | 960 | 40.8 | 1170 | 45.8 |
|  | 3 | 190 | 3.0 | 350 | 25.0 | 540 | 28.0 |  | | 3 | 200 | 5.1 | 980 | 36.0 | 1180 | 41.1 |
| 12 | 1 | 180 | 4.6 | 320 | 31.1 | 500 | 35.7 |  | | 1 | 260 | 7.3 | 890 | 55.4 | 1150 | 62.7 |
|  | 2 | 160 | 3.6 | 200 | 33.8 | 360 | 37.4 |  | | 2 | 250 | 6.6 | 700 | 48.5 | 950 | 55.1 |
|  | 3 | 250 | 2.4 | 320 | 31.8 | 570 | 34.2 |  | | 3 | 260 | 5.7 | 530 | 48.5 | 790 | 54.2 |
| 13 | 1 | 210 | 2.5 | 320 | 27.5 | 530 | 30.0 |  | | 1 | 250 | 5.6 | 580 | 38.5 | 830 | 44.1 |
|  | 2 | 240 | 2.0 | 240 | 32.1 | 480 | 34.1 |  | | 2 | 230 | 4.3 | 560 | 37.1 | 790 | 41.4 |
|  | 3 | 230 | 2.7 | 240 | 30.3 | 470 | 33.0 |  | | 3 | 240 | 3.8 | 420 | 37.2 | 660 | 41.0 |
| 14 | 1 | 160 | 2.0 | 340 | 34.0 | 500 | 36.0 |  | | 1 | 190 | 4.9 | 620 | 40.1 | 810 | 45.0 |
|  | 2 | 180 | 2.8 | 260 | 31.4 | 440 | 34.2 |  | | 2 | 200 | 4.5 | 580 | 39.4 | 780 | 43.9 |
|  | 3 | 160 | 2.3 | 290 | 29.1 | 450 | 31.4 |  | | 3 | 190 | 3.7 | 570 | 41.1 | 760 | 44.8 |
| 15 | 1 | 180 | 1.6 | 250 | 34.0 | 430 | 35.6 |  | | 1 | 250 | 12.1 | 400 | 37.6 | 650 | 49.7 |
|  | 2 | 140 | 3.3 | 330 | 32.5 | 470 | 35.8 |  | | 2 | 250 | 10.8 | 360 | 34.0 | 610 | 44.8 |
|  | 3 | 200 | 2.3 | 280 | 31.2 | 480 | 33.5 |  | | 3 | 230 | 8.1 | 360 | 31.2 | 590 | 39.3 |
| 16 | 1 | 180 | 1.2 | 250 | 20.8 | 430 | 22.0 |  | | 1 | 250 | 5.3 | 520 | 34.8 | 770 | 40.1 |
|  | 2 | 150 | 1.1 | 290 | 25.0 | 440 | 26.1 |  | | 2 | 250 | 4.6 | 330 | 30.1 | 580 | 34.7 |
|  | 3 | 120 | 1.2 | 270 | 24.2 | 390 | 25.4 |  | | 3 | 230 | 3.9 | 250 | 29.2 | 480 | 33.1 |
| 17 | 1 | 210 | 2.3 | 230 | 25.7 | 440 | 28.0 |  | | 1 | 270 | 8.0 | 400 | 32.3 | 670 | 40.3 |
|  | 2 | 200 | 2.9 | 260 | 27.9 | 460 | 30.8 |  | | 2 | 270 | 7.7 | 340 | 30.0 | 610 | 37.7 |
|  | 3 | 200 | 2.7 | 240 | 32.1 | 440 | 34.8 |  | | 3 | 280 | 6.7 | 330 | 27.3 | 610 | 34.0 |
| 18 | 1 | 140 | 1.5 | 270 | 30.3 | 410 | 31.8 |  | | 1 | 200 | 5.0 | 410 | 37.8 | 610 | 42.8 |
|  | 2 | 180 | 2.9 | 290 | 30.3 | 470 | 33.2 |  | | 2 | 210 | 3.3 | 360 | 36.3 | 570 | 39.6 |
|  | 3 | 160 | 1.7 | 240 | 24.5 | 400 | 26.2 |  | | 3 | 200 | 3.1 | 310 | 36.1 | 510 | 39.2 |
| 19 | 1 | 190 | 5.6 | 240 | 35.3 | 430 | 40.9 |  | | 1 | 220 | 8.6 | 520 | 51.1 | 740 | 59.7 |
|  | 2 | 210 | 5.2 | 230 | 40.4 | 440 | 45.6 |  | | 2 | 210 | 7.8 | 470 | 42.1 | 680 | 49.9 |
|  | 3 | 190 | 4.0 | 240 | 35.3 | 430 | 39.3 |  | | 3 | 200 | 5.6 | 420 | 37.2 | 620 | 42.8 |
| 20 | 1 | 160 | 2.5 | 280 | 22.6 | 440 | 25.1 |  | | 1 | 200 | 5.6 | 700 | 46.0 | 900 | 51.6 |
|  | 2 | 210 | 2.4 | 260 | 26.7 | 470 | 29.1 |  | | 2 | 220 | 5.7 | 570 | 44.8 | 790 | 50.5 |
|  | 3 | 210 | 1.9 | 280 | 24.3 | 490 | 26.2 |  | | 3 | 190 | 3.8 | 400 | 40.0 | 590 | 43.8 |
| 21 | 1 | 160 | 2.3 | 370 | 28.8 | 530 | 31.1 |  | | 1 | 200 | 3.2 | 400 | 37.2 | 600 | 40.4 |
|  | 2 | 250 | 3.2 | 330 | 29.2 | 580 | 32.4 |  | | 2 | 200 | 4.6 | 350 | 35.3 | 550 | 39.9 |
|  | 3 | 170 | 2.0 | 330 | 31.0 | 500 | 33.0 |  | | 3 | 210 | 2.4 | 370 | 35.2 | 580 | 37.6 |
| 22 | 1 | 210 | 3.5 | 290 | 33.6 | 500 | 37.1 |  | | 1 | 210 | 11.6 | 490 | 28.2 | 700 | 39.8 |
|  | 2 | 230 | 3.7 | 330 | 33.1 | 560 | 36.8 |  | | 2 | 230 | 11.2 | 380 | 28.4 | 610 | 39.6 |
|  | 3 | 200 | 3.2 | 320 | 30.7 | 520 | 33.9 |  | | 3 | 220 | 8.7 | 320 | 23.7 | 540 | 32.4 |
| 23 | 1 | 170 | 3.6 | 240 | 30.4 | 410 | 34.0 |  | | 1 | 240 | 6.5 | 570 | 51.0 | 810 | 57.5 |
|  | 2 | 210 | 3.2 | 240 | 28.0 | 450 | 31.2 |  | | 2 | 230 | 6.6 | 340 | 49.8 | 570 | 56.4 |
|  | 3 | 180 | 2.9 | 230 | 29.5 | 410 | 32.4 |  | | 3 | 210 | 4.8 | 320 | 47.0 | 530 | 51.8 |
| 24 | 1 | 170 | 2.3 | 260 | 27.0 | 430 | 29.3 |  | | 1 | 280 | 8.2 | 400 | 48.7 | 680 | 56.9 |
|  | 2 | 180 | 1.6 | 250 | 32.0 | 430 | 33.6 |  | | 2 | 230 | 6.4 | 370 | 42.7 | 600 | 49.1 |
|  | 3 | 200 | 1.7 | 250 | 30.9 | 450 | 32.6 |  | | 3 | 240 | 6.7 | 340 | 39.8 | 580 | 46.5 |
| 25 | 1 | 200 | 2.8 | 320 | 33.3 | 520 | 36.1 |  | | 1 | 210 | 7 | 490 | 43.8 | 700 | 50.8 |
|  | 2 | 260 | 3.5 | 310 | 31.8 | 570 | 35.3 |  | | 2 | 240 | 5.7 | 440 | 40.2 | 680 | 45.9 |
|  | 3 | 220 | 3.2 | 330 | 33.0 | 550 | 36.2 |  | | 3 | 250 | 4.3 | 490 | 36.1 | 740 | 40.4 |
| 26 | 1 | 80 | 1.4 | 450 | 30.5 | 530 | 31.9 |  | | 1 | 190 | 3.2 | 960 | 52.0 | 1150 | 55.2 |
|  | 2 | 80 | 1.3 | 400 | 32.2 | 480 | 33.5 |  | | 2 | 180 | 1.8 | 920 | 47.2 | 1100 | 49.0 |
|  | 3 | 70 | 1.2 | 450 | 28.1 | 520 | 29.3 |  | | 3 | 190 | 2.0 | 730 | 43.8 | 920 | 45.8 |
| 27 | 1 | 190 | 1.6 | 300 | 29.1 | 490 | 30.7 |  | | 1 | 240 | 4.2 | 680 | 45.7 | 920 | 49.9 |
|  | 2 | 200 | 2.6 | 330 | 30.5 | 530 | 33.1 |  | | 2 | 210 | 3.2 | 400 | 34.9 | 610 | 38.1 |
|  | 3 | 220 | 2.6 | 270 | 28.3 | 490 | 30.9 |  | | 3 | 230 | 2.9 | 320 | 35.3 | 550 | 38.2 |
| 28 | 1 | 270 | 2.4 | 320 | 22.0 | 590 | 24.4 |  | | 1 | 270 | 4 | 700 | 56.5 | 970 | 60.5 |
|  | 2 | 240 | 1.4 | 330 | 24.2 | 570 | 25.6 |  | | 2 | 250 | 3.6 | 430 | 49.2 | 680 | 52.8 |
|  | 3 | 260 | 1.7 | 360 | 25.1 | 620 | 26.8 |  | | 3 | 230 | 2.7 | 460 | 44.6 | 690 | 47.3 |
| 29 | 1 | 120 | 2.0 | 330 | 22.4 | 450 | 24.4 |  | | 1 | 270 | 3.8 | 650 | 36.5 | 920 | 40.3 |
|  | 2 | 160 | 2.4 | 350 | 25.5 | 510 | 27.9 |  | | 2 | 260 | 3.4 | 620 | 34.8 | 880 | 38.2 |
|  | 3 | 140 | 2.1 | 320 | 24.6 | 460 | 26.7 |  | | 3 | 240 | 2.8 | 520 | 28.5 | 760 | 31.3 |
| 30 | 1 | 220 | 1.4 | 330 | 29.6 | 550 | 31.0 |  | | 1 | 280 | 5.8 | 840 | 51.2 | 1120 | 57.0 |
|  | 2 | 240 | 1.2 | 320 | 27.8 | 560 | 29.0 |  | | 2 | 240 | 5.6 | 590 | 49.6 | 830 | 55.2 |
|  | 3 | 250 | 1.7 | 280 | 26.9 | 530 | 28.6 |  | | 3 | 250 | 4.9 | 490 | 44.7 | 740 | 49.6 |
| 31 | 1 | 180 | 2.0 | 380 | 28.7 | 560 | 30.7 |  | | 1 | 240 | 3.4 | 510 | 33.9 | 750 | 37.3 |
|  | 2 | 220 | 2.6 | 330 | 25.8 | 550 | 28.4 |  | | 2 | 260 | 2.9 | 410 | 33.9 | 670 | 36.8 |
|  | 3 | 220 | 2.8 | 340 | 28.8 | 560 | 31.6 |  | | 3 | 250 | 2.9 | 330 | 32.7 | 580 | 35.6 |
| 32 | 1 | 180 | 2.2 | 430 | 29.2 | 610 | 31.4 |  | | 1 | 230 | 2.7 | 680 | 35.5 | 910 | 38.2 |
|  | 2 | 180 | 2.5 | 410 | 27.7 | 590 | 30.2 |  | | 2 | 230 | 2.8 | 610 | 32.1 | 840 | 34.9 |
|  | 3 | 190 | 1.7 | 410 | 30.4 | 600 | 32.1 |  | | 3 | 230 | 2.6 | 540 | 31.7 | 770 | 34.3 |
| 33 | 1 | 150 | 1.3 | 540 | 24.5 | 690 | 25.8 |  | | 1 | 250 | 2.3 | 420 | 36.3 | 670 | 38.6 |
|  | 2 | 130 | 1.1 | 540 | 26.1 | 670 | 27.2 |  | | 2 | 200 | 1.9 | 410 | 32.0 | 610 | 33.9 |
|  | 3 | 140 | 1.6 | 520 | 24.3 | 660 | 25.9 |  | | 3 | 250 | 1.8 | 360 | 26.5 | 610 | 28.3 |
| 34 | 1 | 230 | 2.6 | 370 | 31.9 | 600 | 34.5 |  | | 1 | 270 | 3.9 | 700 | 41.8 | 970 | 45.7 |
|  | 2 | 230 | 2.3 | 380 | 33.3 | 610 | 35.6 |  | | 2 | 240 | 3.4 | 670 | 32.8 | 910 | 36.2 |
|  | 3 | 210 | 3.7 | 380 | 28.8 | 590 | 32.5 |  | | 3 | 210 | 2.9 | 500 | 29.6 | 710 | 32.5 |
| 35 | 1 | 140 | 2.0 | 410 | 30.2 | 550 | 32.2 |  | | 1 | 290 | 5.2 | 550 | 39.5 | 840 | 44.7 |
|  | 2 | 160 | 2.1 | 370 | 28.3 | 530 | 30.4 |  | | 2 | 240 | 5.3 | 450 | 39.7 | 690 | 45.0 |
|  | 3 | 170 | 2.3 | 420 | 32.6 | 590 | 34.9 |  | | 3 | 250 | 5.2 | 260 | 32.2 | 510 | 37.4 |
| 36 | 1 | 220 | 2.4 | 270 | 26.4 | 490 | 28.8 |  | | 1 | 260 | 7.5 | 640 | 36.8 | 900 | 44.3 |
|  | 2 | 190 | 2.3 | 260 | 24.9 | 450 | 27.2 |  | | 2 | 240 | 4.7 | 500 | 34.0 | 740 | 38.7 |
|  | 3 | 170 | 3.4 | 300 | 25.4 | 470 | 28.8 |  | | 3 | 260 | 3.4 | 440 | 31.5 | 700 | 34.9 |
| 37 | 1 | 150 | 2.6 | 340 | 30.4 | 490 | 33.0 |  | | 1 | 280 | 7.3 | 550 | 39.0 | 830 | 46.3 |
|  | 2 | 160 | 2.0 | 290 | 27.1 | 450 | 29.1 |  | | 2 | 280 | 6.9 | 450 | 39.3 | 730 | 46.2 |
|  | 3 | 180 | 1.9 | 310 | 29.7 | 490 | 31.6 |  | | 3 | 310 | 5.2 | 360 | 36.5 | 670 | 41.7 |
| 38 | 1 | 200 | 2.1 | 310 | 22.6 | 510 | 24.7 |  | | 1 | 280 | 6.9 | 720 | 52.5 | 1000 | 59.4 |
|  | 2 | 190 | 2.1 | 350 | 20.2 | 540 | 22.3 |  | | 2 | 260 | 4.7 | 560 | 48.9 | 820 | 53.6 |
|  | 3 | 180 | 2.0 | 350 | 22.2 | 530 | 24.2 |  | | 3 | 250 | 4.1 | 510 | 45.2 | 760 | 49.3 |

T1: Time to peak anterior CoP displacement; P1: Peak anterior CoP displacement; T2: Time to peak posterior CoP displacement; P2: Peak posterior CoP displacement; T1-2: Time from peak anterior to peak posterior CoP displacement; P1-2: Peak anterior to peak posterior CoP displacement
