# Supplementary material for: The association of reactive balance control and spinal curvature under lumbar muscle fatigue
Source: PeerJ. 2021 Aug 10;9:e11969. doi: 10.7717/peerj.11969 (PMC8362667; doi:10.7717/peerj.11969)
Supplement: Supplemental Information 3 [file peerj-09-11969-s003.docx]

**Table 2.**

Anthropometric parameters and spinal values of the thoracic and lumbar curvatures, sacral tilt and trunk inclination in degrees (°).

|  | | | | **Standing posture** | | | | **Matthiass test without an additional load** | | | | **Matthiass test with 2kg in pre lumbar fatigue** | | | | **Matthiass test with 2kg in post lumbar fatigue** | | | |
| --- | --- | --- | --- | --- | --- | --- | --- | --- | --- | --- | --- | --- | --- | --- | --- | --- | --- | --- | --- |
| **n** | **height (cm)** | **weight (kg)** | **age (years)** | **TK** | **LL** | **ST** | **TI** | **TK** | **LL** | **ST** | **TI** | **TK** | **LL** | **ST** | **TI** | **TK** | **LL** | **ST** | **TI** |
| 1 | 181 | 78 | 21 | 50 | -18 | 2 | -4 | 52 | -27 | 4 | -9 | 50 | -27 | 4 | -10 | 41 | -22 | 3 | -8 |
| 2 | 175 | 90 | 19 | 32 | -21 | 6 | -2 | 25 | -23 | 3 | -10 | 24 | -23 | 6 | -7 | 11 | -20 | 1 | -14 |
| 3 | 187 | 86 | 20 | 38 | -24 | 12 | 2 | 38 | -26 | 10 | -4 | 44 | -26 | 9 | -2 | 35 | -23 | 12 | 1 |
| 4 | 184 | 75 | 20 | 48 | -36 | 19 | 0 | 37 | -33 | 18 | -3 | 40 | -32 | 13 | -6 | 37 | -32 | 16 | -4 |
| 5 | 180 | 71 | 19 | 33 | -29 | 15 | -2 | 25 | -25 | 15 | -3 | 15 | -26 | 16 | -3 | 23 | -21 | 13 | 0 |
| 6 | 188 | 73 | 19 | 22 | -23 | 17 | 1 | 17 | -28 | 17 | -5 | 18 | -32 | 17 | -8 | 14 | -32 | 15 | -12 |
| 7 | 186 | 84 | 23 | 45 | -26 | 12 | -2 | 35 | -22 | 4 | -8 | 36 | -27 | 6 | -7 | 30 | -30 | 9 | -9 |
| 8 | 174 | 76 | 20 | 29 | -25 | 16 | 0 | 26 | -24 | 13 | -3 | 27 | -23 | 12 | -3 | 25 | -19 | 9 | -3 |
| 9 | 198 | 97 | 19 | 30 | -20 | 6 | -5 | 28 | -20 | 4 | -7 | 36 | -24 | 1 | -11 | 38 | -24 | 0 | -10 |
| 10 | 178 | 65 | 20 | 41 | -25 | 9 | -2 | 37 | -27 | 9 | -5 | 22 | -26 | 10 | -9 | 18 | -24 | 8 | -8 |
| 11 | 183 | 85 | 20 | 49 | -35 | 18 | -2 | 50 | -34 | 16 | -3 | 45 | -32 | 13 | -6 | 43 | -30 | 13 | -5 |
| 12 | 187 | 85 | 24 | 51 | -33 | 15 | 1 | 34 | -37 | 17 | -7 | 34 | -35 | 16 | -6 | 35 | -33 | 9 | -10 |
| 13 | 180 | 80 | 19 | 33 | -31 | 16 | -3 | 26 | -28 | 12 | -6 | 20 | -29 | 12 | -11 | 20 | -29 | 13 | -9 |
| 14 | 176 | 72 | 20 | 26 | -22 | 10 | -4 | 22 | -21 | 6 | -9 | 21 | -25 | 8 | -11 | 28 | -25 | 5 | -12 |
| 15 | 181 | 79 | 20 | 19 | -20 | 9 | -5 | 15 | -22 | 6 | -12 | 5 | -17 | 3 | -12 | 15 | -20 | 1 | -14 |
| 16 | 182 | 75 | 20 | 33 | -18 | 9 | -2 | 22 | -17 | 7 | -6 | 21 | -19 | 7 | -8 | 26 | -11 | 2 | -5 |
| 17 | 186 | 82 | 20 | 36 | -23 | 13 | 1 | 34 | -24 | 10 | -4 | 40 | -29 | 11 | -5 | 36 | -19 | 15 | 7 |
| 18 | 183 | 83 | 20 | 24 | -29 | 22 | 3 | 20 | -29 | 20 | -1 | 20 | -30 | 21 | -2 | 9 | -24 | 20 | 1 |
| 19 | 189 | 97 | 20 | 52 | -43 | 21 | -2 | 47 | -39 | 13 | -9 | 53 | -40 | 11 | -9 | 53 | -25 | -3 | -7 |
| 20 | 182 | 80 | 20 | 34 | -24 | 10 | 0 | 28 | -28 | 7 | -8 | 24 | -30 | 8 | -10 | 26 | -32 | 3 | -13 |
| 21 | 184 | 90 | 19 | 18 | -21 | 14 | -2 | 27 | -24 | 12 | -3 | 27 | -26 | 12 | -6 | 22 | -21 | 7 | -7 |
| 22 | 183 | 83 | 18 | 42 | -16 | 5 | 3 | 36 | -20 | 6 | -2 | 43 | -22 | 5 | -2 | 49 | -18 | 5 | 2 |
| 23 | 172 | 72 | 20 | 42 | -35 | 16 | -5 | 27 | -30 | 12 | -9 | 26 | -27 | 8 | -10 | 29 | -28 | 9 | -8 |
| 24 | 188 | 93 | 19 | 44 | -41 | 20 | -6 | 43 | -35 | 13 | -8 | 34 | -35 | 13 | -11 | 39 | -28 | 12 | -4 |
| 25 | 180 | 75 | 21 | 42 | -22 | 11 | -2 | 34 | -28 | 9 | -12 | 43 | -25 | 6 | -10 | 34 | -23 | 2 | -13 |
| 26 | 175 | 64 | 20 | 34 | -33 | 18 | -3 | 27 | -34 | 17 | -7 | 33 | -39 | 18 | -9 | 30 | -36 | 15 | -8 |
| 27 | 180 | 72 | 20 | 36 | -29 | 15 | -3 | 26 | -28 | 14 | -7 | 26 | -30 | 9 | -10 | 26 | -26 | 10 | -6 |
| 28 | 177 | 72 | 22 | 50 | -40 | 21 | -2 | 49 | -37 | 14 | -8 | 44 | -38 | 15 | -8 | 46 | -33 | 16 | 0 |
| 29 | 173 | 69 | 22 | 45 | -31 | 17 | -1 | 33 | -33 | 12 | -9 | 29 | -36 | 15 | -11 | 42 | -32 | 11 | -7 |
| 30 | 183 | 81 | 22 | 40 | -32 | 16 | -1 | 24 | -37 | 15 | -9 | 24 | -40 | 16 | -12 | 26 | -34 | 14 | -11 |
| 31 | 180 | 85 | 21 | 21 | -13 | 5 | -2 | 25 | -10 | 1 | -4 | 10 | -10 | 3 | -5 | 12 | -9 | 3 | -3 |
| 32 | 180 | 91 | 23 | 56 | -21 | 5 | 1 | 43 | -31 | 2 | -14 | 39 | -36 | 4 | -17 | 45 | -26 | -1 | -12 |
| 33 | 180 | 70 | 20 | 39 | -27 | 13 | -5 | 34 | -27 | 10 | -10 | 32 | -24 | 7 | -9 | 35 | -22 | 7 | -6 |
| 34 | 178 | 82 | 21 | 38 | -23 | 9 | -4 | 35 | -26 | 6 | -10 | 34 | -29 | 9 | -11 | 29 | -26 | 9 | -10 |
| 35 | 172 | 70 | 21 | 25 | -23 | 18 | 2 | 14 | -29 | 16 | -8 | 21 | -33 | 16 | -11 | 18 | -28 | 11 | -11 |
| 36 | 182 | 82 | 21 | 47 | -18 | 1 | -4 | 55 | -22 | -5 | -9 | 49 | -25 | -4 | -13 | 56 | -24 | -4 | -13 |
| 37 | 172 | 79 | 21 | 46 | -36 | 22 | 1 | 36 | -33 | 18 | -4 | 43 | -33 | 18 | -3 | 43 | -29 | 16 | 2 |
| 38 | 182 | 66 | 21 | 29 | -20 | 10 | -3 | 26 | -25 | 9 | -9 | 42 | -24 | 5 | -8 | 45 | -24 | 7 | -4 |

TK: thoracic kyphosis; LL: lumbar lordosis; ST: sacral tilt; TI: trunk inclination
